# Research in tree genetic engineering; the Canadian context for field trial of GM trees

**DOI:** 10.1186/1753-6561-5-S7-O62

**Published:** 2011-09-13

**Authors:** Armand Séguin

**Affiliations:** 1 Natural Resources Canada, Canadian Forest Service, Laurentian Forestry Centre, Canada

## 

Canada is in a unique position for the regulation of GM (genetically modified) plants since it is based on the novelty of the resulting traits and not on the process to obtain it. For this reasons the term “plant with novel traits” (or PNTs) is used and these traits can be introduced into plants using biotechnology, mutagenesis or conventional breeding techniques. In Canada, less than a handful of GE tree field trials have been conducted. In all cases, poplars or spruces were used. Consistent with international guidance provided for living modified organisms, particular attention is given to a specific trait by considering both its novelty for the Canadian environment and its potential effect on human health and the environment. Our research is in line with the development of a consolidated research approach and knowledge needed for international guidance (e.g. Cartagena Protocol on Biosafety under the Convention on Biological Diversity) developed for genetically modified organisms. Important losses due to insects and fungal pathogens result in a considerable reduction of yield in Canadian forest productivity. Compounding these problems is the increased movement of pests that has been facilitated by increased international trade of agricultural and forest products as well as global climate change. More recently the increased need for biomass production and specific bioproducts to sustain crop-based biofuel production has emerged. Forest biotechnology has the potential to maintain forest sustainability by contributing to the transformation of our forest industry so as to improve productivity in the context of tree plantation. In this presentation, we will show how genetic engineering of forest trees has been used in improving tree adaptation and pest protection. We will also describe the work being done on environmental assessment of field-grown trees and results on long-term expression of engineered genes.

